# Phylogenetic Relationships of the Marine Haplosclerida (Phylum Porifera) Employing Ribosomal (28S rRNA) and Mitochondrial (*cox1*, *nad1*) Gene Sequence Data

**DOI:** 10.1371/journal.pone.0024344

**Published:** 2011-09-13

**Authors:** Niamh E. Redmond, Jean Raleigh, Rob W. M. van Soest, Michelle Kelly, Simon A. A. Travers, Brian Bradshaw, Salla Vartia, Kelly M. Stephens, Grace P. McCormack

**Affiliations:** 1 Department of Invertebrate Zoology, National Museum of Natural History, Smithsonian Institution, Washington D. C., United States of America; 2 Zoology, National University of Ireland, Galway, Ireland; 3 Zoological Museum, University of Amsterdam, Amsterdam, The Netherlands; 4 National Centre for Aquatic Biodiversity and Biosecurity, National Institute of Water and Atmospheric Research, Auckland, New Zealand; 5 South African National Bioinformatics Institute, University of Western Cape, Bellville, South Africa; University of Melbourne, Australia

## Abstract

The systematics of the poriferan Order Haplosclerida (Class Demospongiae) has been under scrutiny for a number of years without resolution. Molecular data suggests that the order needs revision at all taxonomic levels. Here, we provide a comprehensive view of the phylogenetic relationships of the marine Haplosclerida using many species from across the order, and three gene regions. Gene trees generated using 28S rRNA, *nad1* and *cox1* gene data, under maximum likelihood and Bayesian approaches, are highly congruent and suggest the presence of four clades. Clade A is comprised primarily of species of *Haliclona* and *Callyspongia*, and clade B is comprised of *H. simulans* and *H. vansoesti* (Family Chalinidae), *Amphimedon queenslandica* (Family Niphatidae) and *Tabulocalyx* (Family Phloeodictyidae), Clade C is comprised primarily of members of the Families Petrosiidae and Niphatidae, while Clade D is comprised of *Aka* species. The polyphletic nature of the suborders, families and genera described in other studies is also found here.

## Introduction

Haplosclerid sponges are extremely important in terms of numbers and diversity of species and habitats, as ecosystems, and as producers of bioactive compounds [1]–[4]. Taxonomically, they are also one of the most difficult and unstable groups of the Class Demospongiae *sensu stricto* [5] and a sound classification of the order is a long way from being established. This is because of low numbers of synapomorphies, plasticity of morphological characters, large number of species, and major discrepancies between morphological and molecular data. In the latest complete classification of the Porifera, *Systema Porifera: A guide to the classification of sponges*, the Order Haplosclerida Topsent, 1928 comprises three suborders; Haplosclerina Topsent, 1928, Petrosina Boury-Esnault and Van Beveren, 1982 and Spongillina Manconi and Pronzato, 2002; [6]–[8]. However, molecular data from ribosomal and mitochondrial genes and mitochondrial genomes have shown freshwater sponges (Suborder Spongillina) as closely allied to other demosponge orders including Poecilosclerida and Agelasida, [5], [9]–[13], while nuclear protein coding data is consistent with a monophyletic Haplosclerida [14].

The taxonomic history of the marine species is complicated and many classification schemes have been proposed, [15]–[20]. In *Systema Porifera* [21], [22] each of the two marine haplosclerid suborders, i.e. Haplosclerina and Petrosina, were defined on the basis of skeletal architecture and reproductive strategy (e.g. members of the Haplosclerina are viviparous while those in the suborder Petrosina are oviparous), but they were also seen as being related due to similarities in spicule form and size and their shared chemistry [2], [23]. The monophyly of the marine haplosclerids has been confirmed in a number of molecular-based studies but the monophyly of the each of the two suborders, has been questioned suggesting that reproductive mode is not a good indicator of phylogenetic relationships for this group of sponges [5], [9], [10], [24]. van Soest and Hooper [21], [22] had already suggested that morphological synapomorphies supporting Haplosclerina and Petrosina as suborders were “vague and elusive, many of them being shared by sponges in other groups”, thus it may not be a surprise to find them to be polyphyletic.

Within the suborder Haplosclerina, the secondary ectosomal reticulation described by de Laubenfels [25] characterizes the family Callyspongiidae and should be enough to separate it from the Chalinidae and Niphatidae [18], [26]. While Gray [27] and Lendenfeld [28] suggested that the Chalinidae contained highly unrelated sponges, De Weerdt [29] took the opposite view and collapsed 27 chalinid genera to four and assigned six subgenera to *Haliclona* [30]. Raleigh et al. [24] employing the Erpenbeck fragment of *cox1* [31] suggested that neither of the families Callyspongiidae and Chalinidae nor the genera *Callyspongia* and *Haliclona* were monophyletic. Difficulties at the species level for some *Haliclona* have also been indicated e.g. *H. oculata* and *H. cinerea*, [24], [32].

The molecular evolution of the Haplosclerida has been described as ‘enigmatic’ because their ribosomal genes appear to evolve at a different rate and in a different manner to other demosponges [33], [34] and also the mitochondrial genome of the target species of the sponge genome project (*Amphimedon queenslandica,* Family Niphatidae) has a number of features separating it from the mitochondrial genomes of other demosponges [35]. In this paper we further investigate the phylogenetic relationships in the marine members of this group using DNA sequences of the D1–D5 region of the 28S rRNA from a wide range of marine haplosclerid taxa and additional evidence from mitochondrial data (the Folmer fragment of *cox1* and *nad1*) while also exploring the evolution of these gene regions in haplosclerid taxa.

## Materials and Methods

### Specimens and DNA Extraction

Sponge specimens included in this study were acquired either from the Zoological Museum Amsterdam (ZMA), from the National Institute of Water and Atmospheric Research (NIWA), New Zealand, collected fresh by SCUBA by Dr. Marieke Koopmans (formerly of Wageningen University Research Centre, Netherlands), by Dr. Bernard Picton (National Museums Northern Ireland, UK), by the Biological Institute on Helgoland (BAH) in Germany, or collected in Ireland by Dr. Niamh Redmond and Dr. Grace McCormack. Details of all specimens are listed in Table S1. All specimens had been stored in 100% ethanol and/or 6 M-guanidinium chloride. In the majority of cases, DNA was extracted from the specimens by standard phenol-chloroform-isoamyl extraction followed by ethanol precipitation, otherwise the QIAGEN DNeasy™ Tissue kit was used. Extracted DNA from haplosclerid samples employed in Nichols [5] was also kindly provided by Scott Nichols. A number of marine haplosclerid sequences generated as part of the Porifera Tree of Life project were also kindly provided to help increase taxon sampling in the present study.

### PCR and DNA Sequencing

All primers used in PCR amplification are shown in Table S2. PCR amplification of the D1 to D5 region of the 28S rRNA gene was attempted in three overlapping fragments. Primers from Folmer et al. [36] were utilised for amplifying the 5′ region of the *cox1*. PCR primers to amplify *nad1* were designed from the mitochondrial genomes of *Callyspongia plicifera, Xestospongia muta, Amphimedon compressa* and *Haliclona implexiformis* [37] using the online primer design program PriFi [38, http://cqi-www.daimi.au.dk/cqi-chili/PriFi/main] and a DNA calculator (http://www.sigma-genosys.com/calc/DNAcalc.asp). All gene fragments were amplified in 50 µl reactions, which comprised 5 µl 10X PCR Buffer (Promega), 10 mM dNTPs (Promega), 2 µM primers and 1 unit of *Taq* Polymerase (Promega and Biolabs). MgCl_2_ concentration ranged from 1.5 mM to 3 mM. The temperature regime for the 28S rRNA and *cox1* genes was an initial denaturation of 94°C for 5 min followed by 30 cycles of 1 min at 94°C, 30 sec at annealing temperature (between 38°C to 50°C depending on the DNA template and primer combination) and 1 min to 1 min 30 sec at 72°C. A final extension step of 5 min at 72°C finished the regime. For the *nad1* gene the temperature regime was 10 mins at 94°C, followed by 30 cycles of 30 sec at 94°C, 45 sec at an annealing temperature of 41°C and 90 sec at 72°C with a final extension step of 10 min at 72°C. All products were viewed on a 1% agarose gel stained with ethidium bromide or syber safe using a UV lightsource. PCR products were gel purified and automatically sequenced in both directions (by MWG-Biotech, Germany). It was not possible to amplify and sequence all gene regions from all specimens but from those that were sequenced, the resulting sequences were assembled into contigs using the SeqMan II software from the Lasergene package (DNASTAR Inc.) and the chromatograms were edited by eye. The fully edited consensus sequences were entered into a BLAST algorithm search [39] to check for possible contamination. All sequences have been deposited in to GenBank (accession numbers JN178944-JN179046 (ribosomal sequences) and JN242192-JN242240 (mitochondrial sequences)). Additional sponge sequences for the various gene regions were downloaded from GenBank and used in phylogenetic analyses.

### Alignments

All multiple sequence alignments (including the additional sequences from previous studies that were submitted to GenBank) were assembled and edited in MacClade 4.0 [40]. The D2 region of the 28S rRNA gene was found to be hyper variable for some of the sequences and sequencing and analyses of this region was subsequently abandoned. Separate D1 (74 haplosclerid sequences) and D3–D5 (53 marine haplosclerid sequences) datasets were created. The full D1 alignment was 390 bp in length and 81 bp were removed due to ambiguous alignment (dataset S3). While the full D3 alignment was 735 bp, the alignment used for analysis was 518 bp (dataset S2). A concatenated dataset, (called D1–D5), was created by joining sequences of the D1 and D3–D5 regions from specimens that had both regions available (39 marine haplosclerids; dataset S1). In six cases the D1–D5 sequence originated from two separate individuals of the same species and these are marked with an asterix on the tree produced. Freshwater sponges were initialy chosen as outgroup for marine haplosclerids for all datasets as some data suggests that they are the closest sister group to the marine haplosclerids and, even if they are not the closet sistergroup, they are a monophyletic group within the G4 clade [5]. However, additional analyses were also carried out on the ribosomal and *cox1* datasets (datasets S4 and S5) using additional sequences from sponges who are part of the G4 clade [5] as outgroups. For the *nad1* alignment sequences from a range of other demosponges were included in the analyses using a *Plakinastrella* sequence (EU237487) as outgroup due to the low numbers of available sequences in GenBank for this region (dataset S6).

### Phylogenetic Analyses

Phylogenetic reconstruction was undertaken under a maximum likelihood framework implemented in RAxML 7.0.3 [41] using the GTR model of DNA substitution with model parameters optimised in RAxML, and with confidence levels estimated using bootstrap resampling (1000 replicates) and in PAUP* 4.0b10 (Sinauer Assoc.) using model parameters estimated by jModelTest [42], [43]. Inference under a Bayesian framework was undertaken using MrBayes 3.1.2 using the GTR substitution model with model parameters optimised by the program [44]–[46]. For each dataset, two runs of over 5 million generations were carried out with sampling every 100 generations. The appropriate burnin value was determined by examining the standard deviation of split frequencies to identify when convergence had occurred. A 50% majority rule consensus tree was constructed from all generations sampled after the burnin. Bayesian trees were also reconstructed using a covarion-like model, which allows substitution rates to vary across the tree.

## Results

Although there were differences in the number and distribution of species between the datasets, overall, the tree topologies for the various gene loci were congruent with sequences falling in similar clades and positions. In addition there were very slight differences between ML and Bayesian analyses and between covarian and non covarian models in Bayesian analyses. The trees from RAxML are shown here along with bootstrap proportions (BP) and the posterior probabilities (PP) from the covarion Bayesian analyses (shown as BP/PP in the trees and remainder of the text).

In all trees there was a well supported clade (Clade A) containing multiple species of *Haliclona* and *Callyspongia*. Outside of this clade were a range of marine haplosclerid taxa from the genera *Petrosia, Oceanapia, Acanthostrongylophora, Amphimedon, Cribrochalina, Niphates* and *Xestospongia*. These sequences did not fall in a single clade and the relationships amongst themselves and with Clade A indicate a high diversity of marine haplosclerids. None of the gene trees supported the monophyly of the two marine suborders (Haplosclerina and Petrosina) or the five marine families examined, i.e. Callyspongiidae, Chalinidae, Niphatidae, Petrosiidae and Phloeodictyidae. A total of 12 genera, *Acanthostrongylophora*, *Amphimedon, Callyspongia, Chalinula, Cribrochalina, Haliclona, Neopetrosia, Niphates, Oceanapia, Petrosia, Siphonochalina* and *Xestospongia* were polyphyletic. The monophyletic status of the genera *Calyx, Cladocroce, Dasychalina, Dendroxea, Gelliodes, Hemigellius, Pachychalina* and *Tabulocalyx* could not be established as there was only one representative of these genera included in each of the various analyses. The genus *Aka* was found to be monophyletic but had few representatives.

### 28S rDNA phylogenies

**Figure 1:**
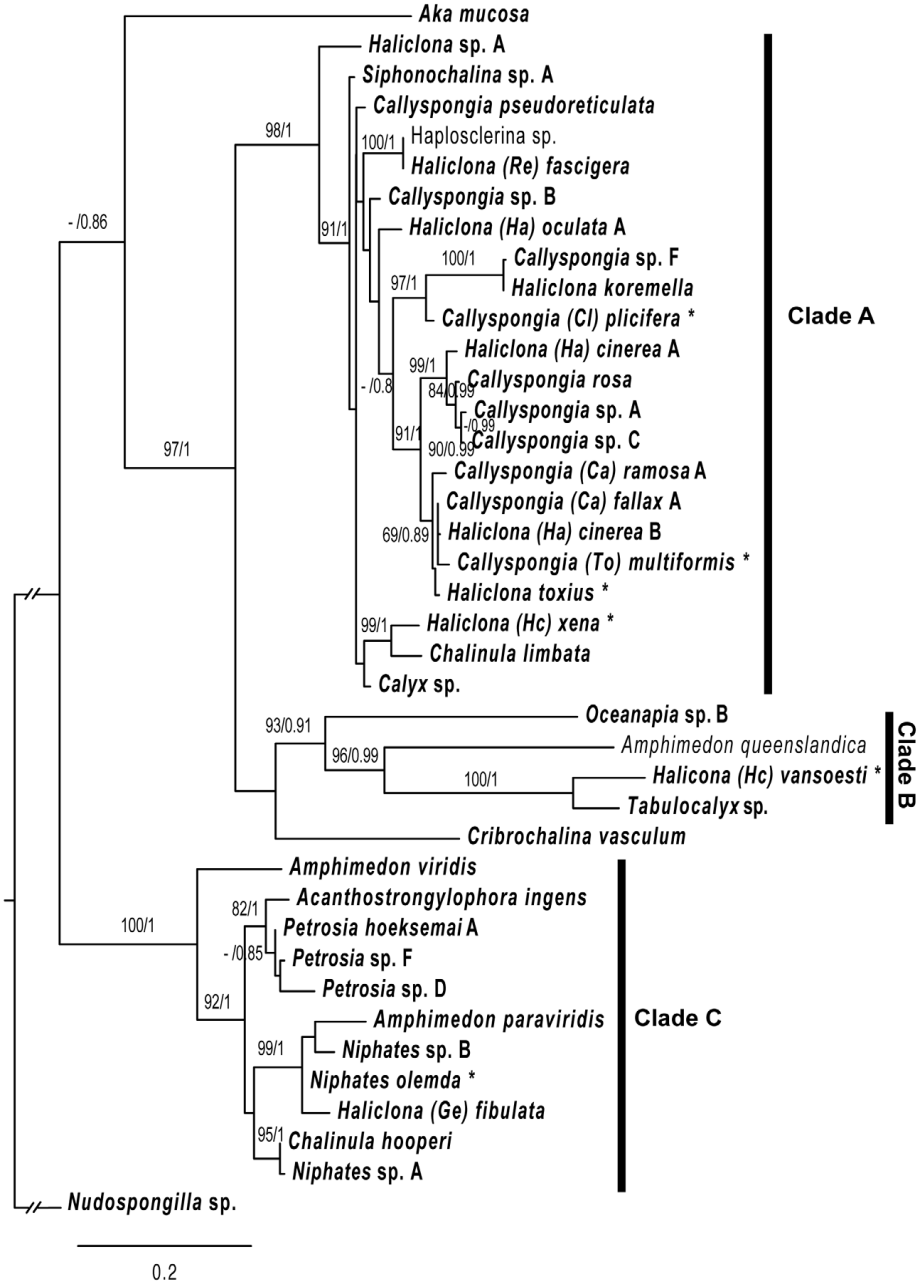
Maximum-likelihood phylogeny reconstructed using the D1–D5 region of the 28S rRNA gene. The DNA substitution model parameters estimated by RAxML were; f(A) 0.24, f(C) 0.24, f(G) 0.33, f(T) 0.19; R(AC) 0.53, R(AG) 1.73, R(AAT) 1.0, R(CG) 0.52, R(CT) 5.71, R(GT) 1.0; alpha 0.52; pinvar 0.43. Sequences produced during this study are in bold. Sampling locations for each taxon are given in Table S1. Other sequences were downloaded from Genbank (*A. queenslandica*, EF654518, Haplosclerina sp., AY561860). Taxon labels showing an * are those comprised of sequences from two specimens, in each case the D3 sequence was downloaded from Genbank (*C. multiformis*, AF441344 *C. plicifera*, AF441345; *H. toxius*, AF441342; *H. vansoesti*, AF441346; *N. olemda*, AF441353 and *H. xena*, AY319327). Numbers on the branches represent bootstrap proportions/posterior probabilities.

The D1–D5 28S rDNA ML phylogeny is presented in Figure 1. This dataset had sequences from 39 marine haplosclerid species included from five families and 13 genera. Clade A was supported by 98/1 (BP/PP) and contained ten *Callyspongia* species, eight *Haliclona* species, *Chalinula limbata,* a *Calyx* sp., a *Siphonochalina* sp. and an individual identified as Haplosclerina sp. [9]. Smaller supported groupings of *Callyspongia* and *Haliclona* species were present within this clade. One such group had 69/0.89 support and contained *H. cinerea* B (POR14110), *H. toxius, C. fallax, C. multiformis and C. ramosa* A (MKB3142). Another clade (99/1) contained three *Callyspongia* species and *H. cinerea* A (POR17651). A third group with 97/1 support contained *C. plicifera, H. koremella* and *Callyspongia* sp. F ((MKB1668). Sequences from *Amphimedon queenslandica, Haliclona vansoesti, Tabulocalyx* sp. and *Oceanapia* sp. B (MKB586) formed another highly supported clade (Clade B, 93/0.91). Long branch lengths for this group suggest undersampling and/or high divergence. *Cribrochalina vasculum* was sister to Clade B but without support. A relationship between Clades A+B and *Cribrochalina vasculum* was also highly supported (97/1).

Within a third clade (Clade C supported by 100/1) three *Petrosia* species clustered with *Acanthostrongylophora ingens* (82/1). Two *Niphates* sequences were present in the same clade with *Haliclona fibulata* and *Amphimedon paraviridis* (99/1). *Chalinula hooperi* clustered with a third *Niphates* (sp. A, POR14462) with 95/1 support rather than with *C. limbata*, which, as mentioned above, was in Clade A. The sequence from *Amphimedon viridis* grouped within this clade rather than with the *A. queenslandica* sequence in Clade B. *Aka mucosa* was in an isolated position.

Tree reconstructions of the 28S rDNA D3–D5 data (Figure S1) included sequences from an additional 14 marine haplosclerid specimens not included in the D1–D5 28S rRNA gene dataset. In Clade A there were additional sequences from *Neopetrosia subtriangularis, Haliclona manglaris* and another *C. fallax*. Clade B (83/0.95) contained the same four taxa as described previously with the addition of new sequences from *Xestospongia caminata* and *Haliclona vermeuleni*. The former was sister to the five other members of the clade and *H. vermeuleni* grouped with *A. queenslandica* with support of 100/1. In Clade C (100/1) the three additional *Amphimedon* sequences (*A. viridis*, AF441350 and *A. compressa*, AF441351 Genbank sequences and a newly generated *A. compressa* sequence (Table S1)) grouped with *A. paraviridis* and the newly added *N. erecta* sequence (94/1) and this clade was positioned distantly from the other *A. viridis* sequence. The additional *Acanthostrongylohora ashmorica* sequence grouped near the *A. ingens* sequence and *Haliclona walentinae* was also present in Clade C. Elsewhere, *A. mucosa* clustered with two congener sequences, *A. coralliphaga* and *Aka* sp., with support of 100/1 (Clade D).

**Figure 2:**
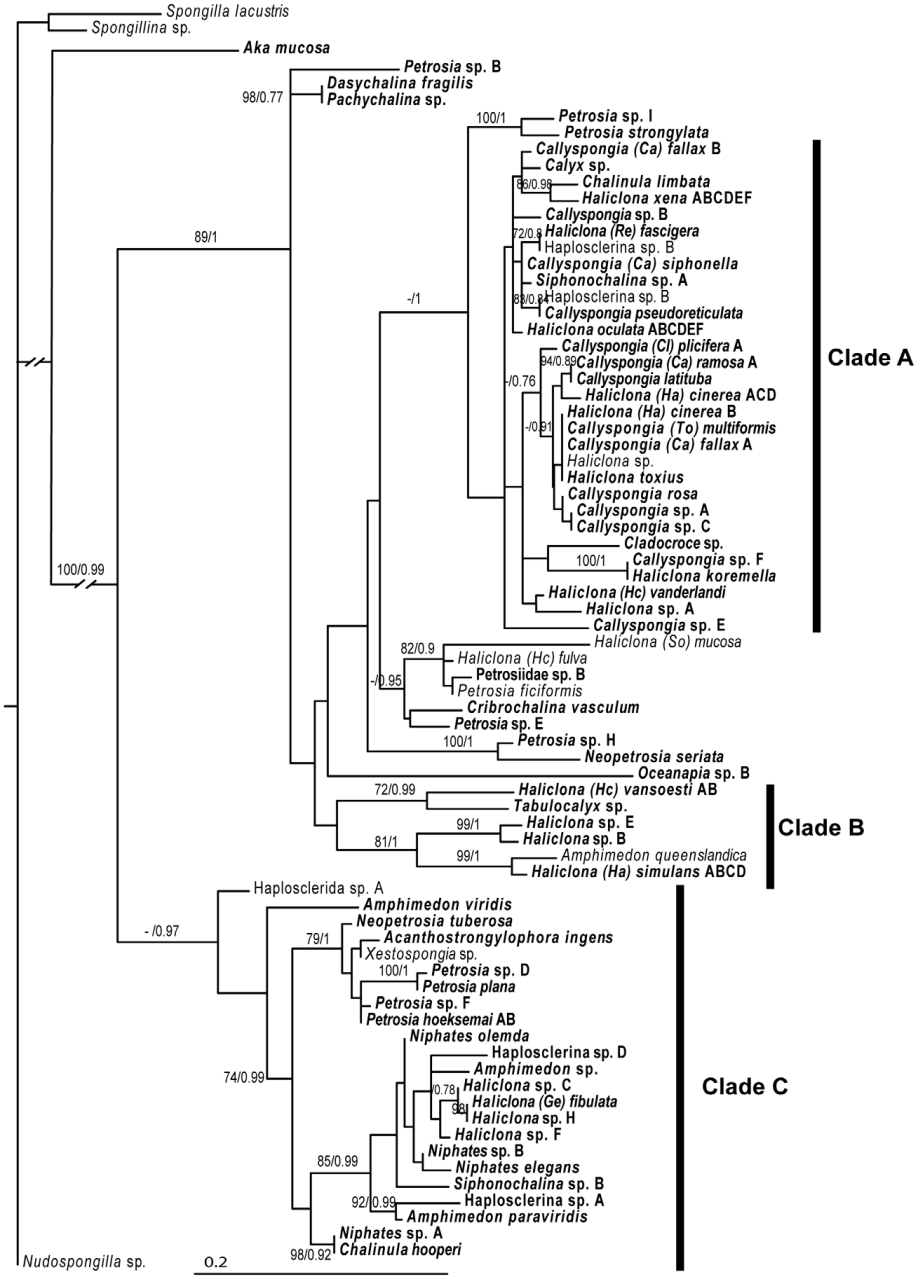
Maximum-likelihood phylogeny reconstructed from the D1 region of the 28S rRNA gene. The DNA substitution model parameters estimated by RAxML were; f(A) 0.26, f(C) 0.24, f(G) 0.33, f(T) 0.17; R(AC) 0.69, R(AG) 2.17, R(AAT) 1.39, R(CG) 0.61, R(CT) 5.12, R(GT) 1.0; alpha 0.61; pinvar 0.36. Sequences produced during this study are in bold. Sampling locations for each taxon are given in Table S1. Other sequences were downloaded from Genbank (Haplosclerida A, AY561856, Haplosclerina C AY561861, Haplosclerina B, AY561860, *Haliclona* sp, AY561862, *H. mucosa*, AJ225831, *H. fulva*, AJ225829, *P. ficiformis*, AJ225828, *A. queenslandica*, EF654518, *Xestospongia* sp., AY561853). Numbers on the branches represent bootstrap proportions/posterior probabilities.

The D1 dataset was the largest and most diverse marine haplosclerid dataset in this study as it contained sequences from 74 marine haplosclerid taxa from five families and 18 genera (Figure 2). However, it was also the shortest alignment being just over 300 bp long after variable bases were removed and should thus be used to give a broad indication of which clade sequences are allied to rather than a reliable picture of relationships, as there was no resolution at many of the internal branches. On the gene trees, the large clade containing subclades A+B+*C. vasculum* was highly supported with 89/1 (Figure 2). Relationships amongst sequences were broadly the same as the two previous datasets. *Cladocroce* is included for the first time in the analyses and was positioned amongst the Clade A sequences. Some of the new sequences grouped with *Cribrochalina vasculum*, such as *Haliclona mucosa, H. fulva, Petrosia ficiformis, Petrosia*. sp. E (MKB1028) and Petrosiidae sp. B (MKB1785) however this grouping only had support in the Bayesian analyses (0.95). In Clade B *A. queenslandica* had 99/1 for a sister relationship with the newly added *H. simulans*, and a further two new *Haliclona* were sister to these two species (81/1). A different *Pachychalina* sp. was sequenced for this region (in comparison to the D3–D5 analyses) and was found in the larger Clade A+ Clade B rather than in Clade C. In Clade C there was high support for the grouping of *Petrosia* sp. D (MKB1020) and the additional *P. plana* sequence (100/1). A second *Siphonochalina* specimen included was positioned in Clade C rather than with the other *Siphonochalina* sp. A (POR14630), which was in Clade A.

### Mitochondrial gene phylogenies

The *cox1* tree reconstructed using a large dataset (containing cnidarian, homoscleromorph and demosponge sequences), shows the presence of very divergent patterns of sequence evolution in the *cox1* across different demosponges (Figure S2). A clade of anthozoan sequences was present within the demosponge clade. The divergent patterns of haplosclerid sequences first shown in Erpenbeck et al. [47] are also visible on this tree, however clades corresponding to those in the ribosomal gene trees are also recovered. There is a clade equivalent to Clade A supported by 96 BP and a number of smaller more divergent clades with high support associated with it. However, positioned distantly from this clade are four *Niphate*s sequences (100 BP) and an additional clade containing *A. queenslandica, H. caerulea, C. vaginalis, C. armigera*, and *H. simulans* (corresponding to Clade B). An *A. compressa* sequence clusters very tightly within the clade of keratose sponges (99 BP). Thus very different sequence patterns are to be found amongst marine haplosclerid species in this gene region. Divergent patterns are also seen within the Poecilosclerida, Hadromerida and Halichondrida.

**Figure 3:**
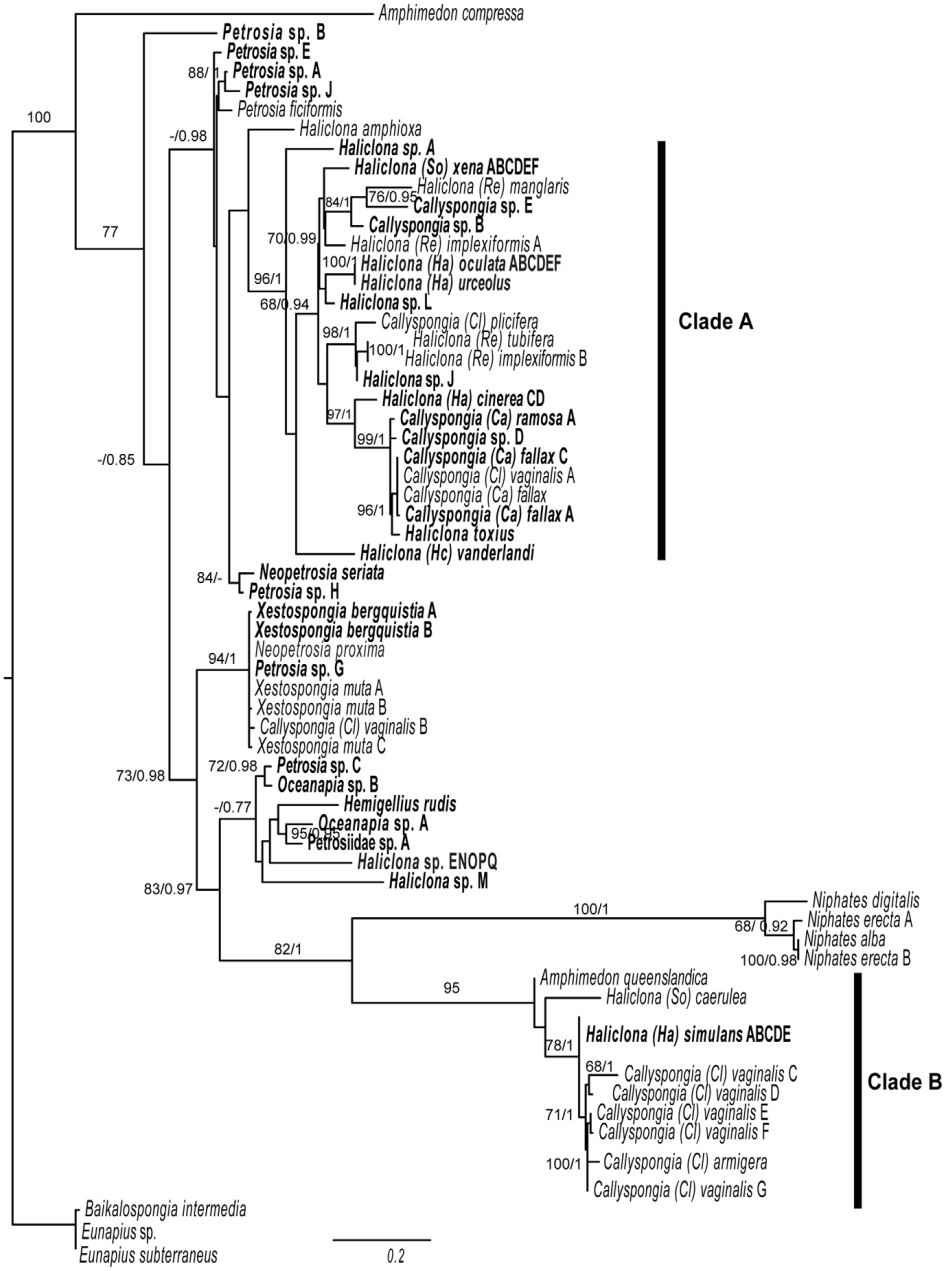
Maximum-likelihood phylogeny reconstructed from the Folmer (5′) region of the *cox1* gene from only Haplosclerida taxa. The DNA substitution model parameters estimated by RAxML were; f(A) 0.26, f(C) 0.15, f(G) 0.22, f(T) 0.37; R(AC) 1.59, R(AG) 3.12, R(AT) 0.86, R(CG) 0.44, R(CT) 5.28, R(GT) 1.0; alpha 0.7; pinvar 0.44. Sequences produced during this study are in bold. Sampling locations for each taxon are given in Table S1. Other sequences were downloaded from Genbank (*A. compressa*, EF519558, *P. ficiformis*, EF519663, *H. amphioxa*, AJ843892, *H. manglaris*, EF519626, *H. implexiformis*, EF519625, *C. plicifera*, EU237477, *H. tubifera*, EF519624, *H. implexiformis* B, EF519623, *C. vaginalis* A, GQ415412, *C. fallax*, GQ415417, *N. proxima*, AM076980, *X. muta* A, EF519699, *X. muta* B, EU716650, *C. vaginalis* B, EF095182, *X. muta* C, EF095185, *N. digitalis*, EF519658, *N. erecta* A, EF519660, *N. erecta* B, EF519659, *N. alba*, EF519654, *A. queenslandica*, DQ915601, *H. caerulea*, EF519619, *C. armigera* EF519578, *C. vaginalis* C–G, EF519577, EF519579, EF519581, GQ304697, GQ304613). Numbers on the branches represent bootstrap proportions/posterior probabilities.

For the dataset that only included all marine haplosclerid *cox 1* sequences, using freshwater sponges as outgroups (Figure 3) we see that the *A. compressa* sequence falls in a basal position and has a long branch. The clade of *Niphate*s sequences (100/1) are possibly attracted by the long branch of the clade containing the *A. queenslandica* sequence and these together have high support (82/1). They happen to fall well inside the marine haplosclerid set of sequences however, as a sister group to a clade containing *Oceanapia* and *Petrosia* sequences (amongst others), which is not unlike the pattern seen in ribosomal trees. There are a number of additional patterns in the *cox*1 data however, that should be mentioned. Firstly, sequences from different species were found to be identical. These included those sequenced as part of the same study i.e. *N. erecta* B (EF519659) & *N. alba* (EF519654), and *H. tubifera* (EF519624)& *H. implexiformis* (EF519623) [47], *C. fallax* (GQ415412) and *C. vaginalis* A (GQ415417) [48] and those sequenced as part of different studies (i.e. *Eunapius* sp. (DQ167181) and *E. subterraneous* (FJ715439) (from Hess et al. (Direct submission in GenBank) and Harcet et al. [49] respectively) and all four sequences from *X. muta* (EF519699) [47], *N. proxima* (AM076980) [50], *X. bergquistia* A & B and *Petrosia* sp. G (this study). The second noteworthy pattern is of very different sequences being returned from the same species. For the case of *C. vaginalis* sequence (GQ415412) from the study of López-Legentil et al. [48] grouped in Clade A with three *C. fallax* sequences, being identical to one of them. A second *C. vaginalis* sequence (EF095182) [11] clustered with *X. bergquistia* while those from Erpenbeck et al. [47] and DeBiasse et al. [51] clustered in the same clade as *A. queenslandica* quite distantly from the rest of the marine haplosclerid sequences (Figure S2). All of the >200 sequences from the study of De Biasse et al. [51] clustered in this position (data not shown).

The *nad1* phylogeny (Figure S3) showed a monophyletic Clade A was retrieved with 100/1 support. Within this clade sequences generated from multiple specimens from *H. cinerea* were almost all identical as were two sequences from *H. oculata* and two from *H. xena*. The same pattern, seen in other gene trees, of smaller clades containing *Haliclona* and *Callyspongia* sequences were also evident on this phylogeny. *A. queenslandica* did not group with *A. compressa* and both had a very long branch. Instead *A. compressa* grouped with *Petrosia plana* (99/1) in what might be Clade C but is very poorly sampled for this locus. The sequence generated from *Xestospongia bergquistia* was very similar to that from *X. muta* (100/0.99) and they grouped together on the tree with two *Haliclona* sp. sequences (88/0.86). The *Dasychalina fragilis* sequence was in an unsupported position.

## Discussion

The gene trees shown here, generated from additional molecular data from three different genes, are highly congruent with phylogenies produced from 18S rDNA data and from data generated from the 3′ (Erpenbeck) region of the *cox1* gene [10], [24]. The data from all four genes (18S and 28S rRNA, *cox1* and *nad1*) suggests the presence of four clades.

The first, Clade A, is highly complex and dominated by species that have been identified as *Haliclona* in Family Chalinidae, and *Callyspongia* in Family Callyspongiidae. A few species of *Chalinula* and *Cladocroce* (Family Chalinidae) are also included in Clade A, along with *Siphonochalina* (Family Callyspongiidae) and a species identified as *Calyx* from the Family Phloeodictyidae. *Haliclona*, *Chalinula*, and *Siphonochalina* also appear in Clades B and C but to a much lesser extent than in Clade A. This clade would appear to be a combination of the two families Chalinidae and Callyspongiidae.

A range of smaller clades comprising species of *Petrosia*, *Acanthostrongylophora*, and *Xestospongia* (Family Petrosiidae), *Amphimedon, Cribrochalina, Niphates* (Family Niphatidae), and *Oceanapia* (Family Phloeodictyidae) were highly variable in their arrangement in relation to Clade A and to each other. Clade B was smaller, and consistently composed of the same two species of *Haliclona, H. simulans* and *H. vansoesti* (Family Chalinidae), *Amphimedon queenslandica* (Family Niphatidae), and a species of *Tabulocalyx* (Family Phloeodictyidae). Clade C differs considerably from Clade A in that it is dominated by species that have been identified as *Petrosia*, *Neopetrosia*, *Xestospongia*, and *Acanthostrongylophora* in the Family Petrosiidae, and *Niphates* and *Amphimedon* in the Family Niphatidae. This clade appears to be a combination of two families Niphatidae and Petrosiidae. A fourth clade was comprised of members of the genus *Aka*, a group of sponges that are presently classified with Family Phloeodictyidae, but which are very different from most Haplosclerida in that they excavate calcareous substrates and are externally visible only by their fistulose tubes.

The relationships between *Haliclona* and *Callyspongia* species and other Chalinidae and Callyspongiidae indicated in Clades A–C are perplexing because the genera are thought to be very clearly defined by morphological characteristics. These two families, with Phloeodictyidae, Niphatidae and Petrosiidae are recognisable along a gradient of decreasing spongin reinforcement (or increasing siliceousness), from Chalinidae to Petrosiidae. These trends are reflected in the overall structure of the molecular phylogenies, with Clade A dominated by Callyspongiidae and Chalinidae, and Clade C, dominated by Petrosiidae and Niphatidae. However, it is clear from these molecular data that the genera that form these families are not as easy to separate as was previously thought. This may be reflected also by the inherently great difficulties in the identification of ‘transitional’ haplosclerid species. In the Indo-west Pacific, for example, there are numerous species that appear to transition between different genera including what we currently define as *Niphates*, *Amphimedon*, *Dascychalina* and *Pachychalina* and the numerous varied forms of supposed Callyspongiidae in this region (MK, Pers. Obs). However, it is encouraging to see molecular data consistently support the two New Zealand species currently recognised under the umbrella genus *Callyspongia* (*C*. *ramosa*, *C*. *latituba*) but which are clearly a new genus or subgenus. Similarly, two species of *Acanthostrongylophora* (*A. ingens*, *A. ashmorica*) previously thought to be an unusual form of *Xestospongia* or *Petrosia*, are also consistently grouped together, and with other Petrosiidae.

It is possible that re-evaluation of genus groups and their associated major biochemical components, including the metabolic pathways of various compounds, may provide additional support for the alternative relationships proposed by molecular data. The use of biochemical compounds for phylogenetic classification has been rejected by a number of authors due to disagreement between biochemical and morphological data [52], [53], but Urban et al. [54] and Hu et al. [55] found that the disagreement between biochemical and morphological data is largely a problem of incorrect taxonomic identification of the sponge from which the compounds were identified. The problem of mis-identification is particularly acute amongst species in genera with a dearth of strong diagnostic morphological characters, and this is particularly so in the Haplosclerida with few megasclere and microsclere forms, and what appears to be a gradient of siliceous and fibrous development. The biochemical observations in marine haplosclerid taxa by Fromont et al. [56] and van Soest et al. [57] are largely congruent with molecular data.

The usefulness of developmental characters, also should not be ruled out. Bergquist et al. [58] distinguished two larval types in the Haplosclerida, one group represented by *Chalinula* and *Reniera* and the other by *Callyspongia*, *Adocia* and *Haliclona*. However, de Weerdt [29] found it difficult to separate adults of *Adocia*, *Haliclona* and *Reniera* so placed them all into the genus *Haliclona* and suggested that differences in larval structure were not enough to separate them and were of minor importance. Re-investigating these larval characteristics may prove worthwhile in light of relationships indicated in this paper. We have shown for example that many *Callyspongia* and *Haliclona* sequences are associated with each other on all gene trees. Furthermore, on the D1 trees *Haliclona* (*Reniera*) *fulva* and *H.* (*Reniera*) *mucosa* sequences group away from this clade.

The Haplosclerida have long been recognised as a highly diverse group [2] and this is clearly demonstrated in this study. Suggestions of the polyphyletic nature of the various taxa within haplosclerids have been appearing in various publications since 2002 [5], [9]–[11], [24], [59] with 18S, partial 28S rRNA genes and mitochondrial data. However, a number of authors have also suggested that the differing nature of the molecular phylogenies (when compared to morphology) may be due to a higher rate of substitution in the ribosomal and mitochondrial genes in marine haplsoclerids when compared to other demopsponges and that as a result they are not suitable for phylogeny reconstruction in this group [11], [33], [47]. Nonetheless, previous studies [9], [11], [33], [59] had included fewer haplosclerid species, and, given the high diversity in the group we are not surprised that branch lengths were longer in some taxa. With the addition of a higher number of sequences and a longer region of 28S rDNA we find that the relationships remain congruent and branch lengths within the haplosclerids are comparable to those in other groups of sponges. Furthermore, Redmond and McCormack [34] showed that the variable indels found in the 18S rRNA gene are very important synapomorphies. Therefore although it may be difficult to use this region to compare haplosclerids with other sponges, due to the indels present in Clade A, 28S rDNA data shows strong phylogenetic signal for studying relationships within the marine Haplosclerida and highlights a very high diversty within this group, which is supported by other data.

Mitochondrial data is not straightforward for haplosclerid phylogenetics. Using the 5′ end of the *cox1* Erpenbeck et al. [47] found the marine Haplosclerida to be polyphyletic in their study of Caribbean demosponges. They pointed out the high evolutionary rate found in the mt genome of *A. queenslandica* [60] and concluded that the *cox1* would not be suitable to resolve Haplosclerida relationships sufficiently. The addition of further sequences through this study certainly suggests that this is true. We would suggest that the positions of the major clades within the order cannot be reliably determined using this locus but support is strong within those clades. In *cox1* and *nad1* gene trees branches leading to the *A. queenslandica* sequence were long but branch lengths leading to most other marine haplosclerid sequences were not. This pattern was also shown in Wang and Lavrov [37] who showed that *A. queenslandica* has an unusual mitochondrial genome, lacking genes, containing deletions in several genes, and displaying an accelerated rate of sequence evolution. Analysis from three additional marine haplosclerid species found no similar features indicating that the *A. queenslandica* mitochondrial genome has undergone unusual evolution and is a poor representative of the G3 clade [37]. Thus, as found in other metazoans (e.g. the nematode *Caenorhabidites elegans*) it is reasonable to suggest that the evolutionary rate in some species within a taxon may be higher than others and therefore inclusion of these taxa may lead to erroneous views of phylogeny [61]. The *cox1* data produced to date for marine haplosclerids has indicated a number of other species have unusual evolutionary patterns in their mitochondrial genomes (e.g. *C. vaginalis*, *N. erecta*, *H. simulans*). It is likely that additional demosponge sequences should also be viewed with caution, including some of the poecilosclerid sequences from Erpenbeck et al. [47] that have exceedingly long branch lengths (e.g. *Mycale* and *Monanchora*, *Chondrosia*, *Chelonaplysila*). Despite the *cox1* gene evolving more slowly in sponges compared to other metazoans it is clear that it is not suitable for reconstructing relationships across an entire order and between orders. There are also clear examples of where the *cox1* data both assists and hinder barcoding by showing different species to be identical and the same species to have very different sequence patterns. It has been shown here and in other studies [24], [34] that specimens identified as *C. vaginalis*, *H. cinerea* and *H. oculata* may comprise of more than one OTU, and there are indications that species that should be closely related are in fact not. The evolution of mitochondrial genomes continues to be an exciting area of research and will offer further illumination in time.

Although all of the four genes employed to date are not all independant (two ribosomal genes and two mitochondrial genes), and there are problems in using these genes for certain taxa within the Haplosclerida, the patterns in all four genes are reasonably congruent lending support to suggestions that the suborders and some of the families and genera should be revised. There is also some support for the molecular phylogenetic patterns, not only from the general trends in morphological data, but also in secondary metabolite and biochemical signals, and possibly in reproductive traits. It is now time to employ alternative approaches to find synapomorphies between taxa suggested as being closely related by molecular data. Further acquisition of ribosomal and mitochondrial data is necessary of species that would potentially fall in the poorly represented clades within the Haplosclerida. Furthermore, data from nuclear protein coding genes would provide additional support for deep branches in the tree, confirm results from ribosomal and mitochondrial genes and help to find the sister group of the Order Haplosclerida.

## Supporting Information

Figure S1 **Maximum-likelihood phylogeny reconstructed from the D3-D5 region of the 28S rRNA gene.** The DNA substitution model parameters by RAxML were; f(A) 0.23, f(C) 0.24, f(G) 0.32, f(T) 0.21; R(AC) 0.57, R(AG) 2.03, R(AAT) 1.0, R(CG) 0.57, R(CT) 4.19, R(GT) 1.0; alpha 0.19. Sequences produced during this study are in bold. Sampling locations for each taxon are given in Table S1. Other sequences were downloaded from Genbank (*A. coralliphaga*, AF441345, *A. queenslandica*, EF654518, Haplosclerina B, AY561860, *C. plicifera*, AF441345, *H. toxius*, AF441342, *H. vansoesti*, AF441346, *N. olemda*, AF441353, *H. xena*, AY319327, *N. subtriangularis*, AF441341, *C. fallax*, AF441344, *X. caminata*, AF441348, *Pachychalina* sp. B, AF441352, *A. viridis*, AF441350, *A. compressa*, AF441351, *A. ashmorica*, AF441354, *H. vansoesti*, AF441346). Numbers on the branches represent bootstrap proportions/posterior probabilities.
(TIF)

Figure S2 **Maximum-likelihood phylogeny reconstructed from the Folmer (5′) region of the** ***cox1*** **gene.** The DNA substitution model parameters estimated by RAxML were; f(A) 0.26, f(C) 0.15, f(G) 0.22, f(T) 0.37; R(AC) 1.37, R(AG) 4.1, R(AT) 1.43, R(CG) 1.18, R(CT) 6.87, R(GT) 1.0; alpha 0.71; pinvar 0.41. Sequences produced during this study are in bold. Sampling locations for each taxon are given in Table S1. Other sequences were downloaded from Genbank. Numbers on the branches represent bootstrap proportions.
(TIF)

Figure S3 **Maximum-likelihood phylogeny reconstructed from the** ***nad1*** **gene.** The DNA substitution model parameters by RAxML were; f(A) 0.3, f(C) 0.1, f(G) 0.21, f(T) 0.39; R(AC) 4.18, R(AG) 6.72, R(AT) 1.0, R(CG) 4.18, R(CT) 11.9, R(GT) 1.0; alpha 0.26; pinvar 0.08. Sequences produced during this study are in bold. Sampling locations for each taxon are given in Table S1. Other sequences were downloaded from Genbank *(P*. cf. *ankodes,* EU237487, *X. muta*, EU237490, *A. queenslandica*, DQ915601, *C. plicifera*, EU237477, *A. compressa*, EU237474, *C. kuekenthali*, EU237479, *G. neptuni,* AY320032, *E. muelleri*, EU237481, *T. actinia*, AY320033, *A. schmidti*, EU237475, *A. corrugata*, AY791693, *H. dujardini*, EU237483, *Chondrilla* sp., EU237478, *T. ophiraphidites*, EU237482). Numbers on the branches represent bootstrap proportions/posterior probabilities.
(TIF)

Table S1 **List of all the marine haplosclerid specimens sequenced in this study.** Sampling location, voucher number where available, and what gene regions were sequenced for each is included.
(DOC)

Table S2 **Primer sequence information for each primer used in amplifying each gene region.**
(DOC)

Dataset S1 The concatenated dataset created by joining sequences of the D1 and D3–D5 regions from specimens that had both regions available.
(NEX)

Dataset S2 The final D3 alignment used for analysis.
(NEX)

Dataset S3 The final D1 alignment used for analysis.
(NEX)

Dataset S4 The *cox1* dataset including sequences from other demospnge orders and cnidarians.
(NEX)

Dataset S5 The *cox1* dataset including sequences from only Haplosclerida.
(NEX)

Dataset S6 The *nad1* alignment used for analysis.
(NEX)
