# Supplementary figures and images for: Phylogenetic Relationships of the Marine Haplosclerida (Phylum Porifera) Employing Ribosomal (28S rRNA) and Mitochondrial (cox1, nad1) Gene Sequence Data
Source: PLoS One. 2011 Sep 13;6(9):e24344. doi: 10.1371/journal.pone.0024344 (PMC3172223; doi:10.1371/journal.pone.0024344)

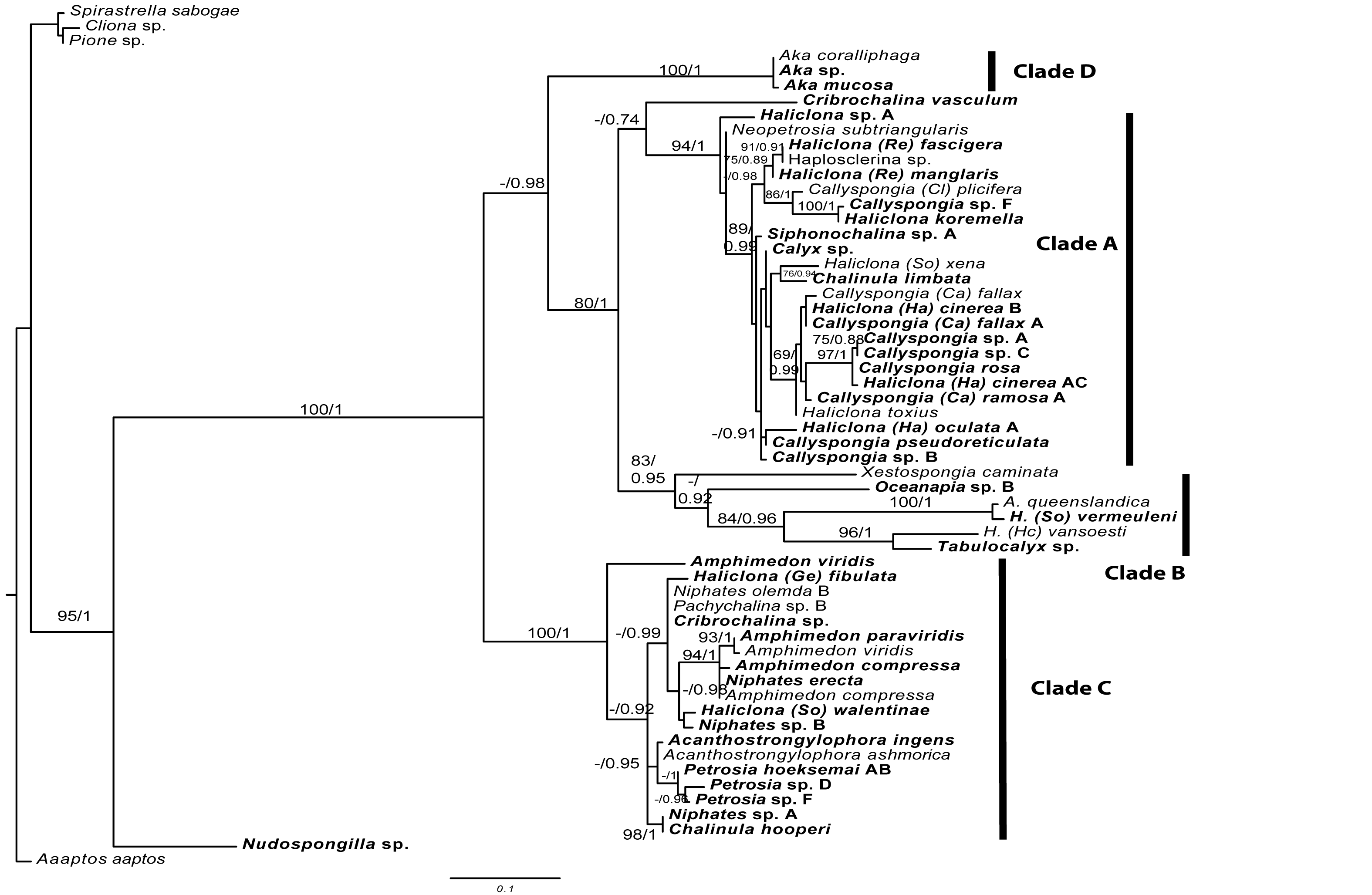

Supplement: Figure S1 — Maximum-likelihood phylogeny reconstructed from the D3-D5 region of the 28S rRNA gene. The DNA substitution model parameters by RAxML were; f(A) 0.23, f(C) 0.24, f(G) 0.32, f(T) 0.21; R(AC) 0.57, R(AG) 2.03, R(AAT) 1.0, R(CG) 0.57, R(CT) 4.19, R(GT) 1.0; alpha 0.19. Sequences produced during this study are in bold. Sampling locations for each taxon are given in Table S1. Other sequences were downloaded from Genbank (A. coralliphaga, AF441345, A. queenslandica, EF654518, Haplosclerina B, AY561860, C. plicifera, AF441345, H. toxius, AF441342, H. vansoesti, AF441346, N. olemda, AF441353, H. xena, AY319327, N. subtriangularis, AF441341, C. fallax, AF441344, X. caminata, AF441348, Pachychalina sp. B, AF441352, A. viridis, AF441350, A. compressa, AF441351, A. ashmorica, AF441354, H. vansoesti, AF441346). Numbers on the branches represent bootstrap proportions/posterior probabilities. (TIF) [file pone.0024344.s001.tif]

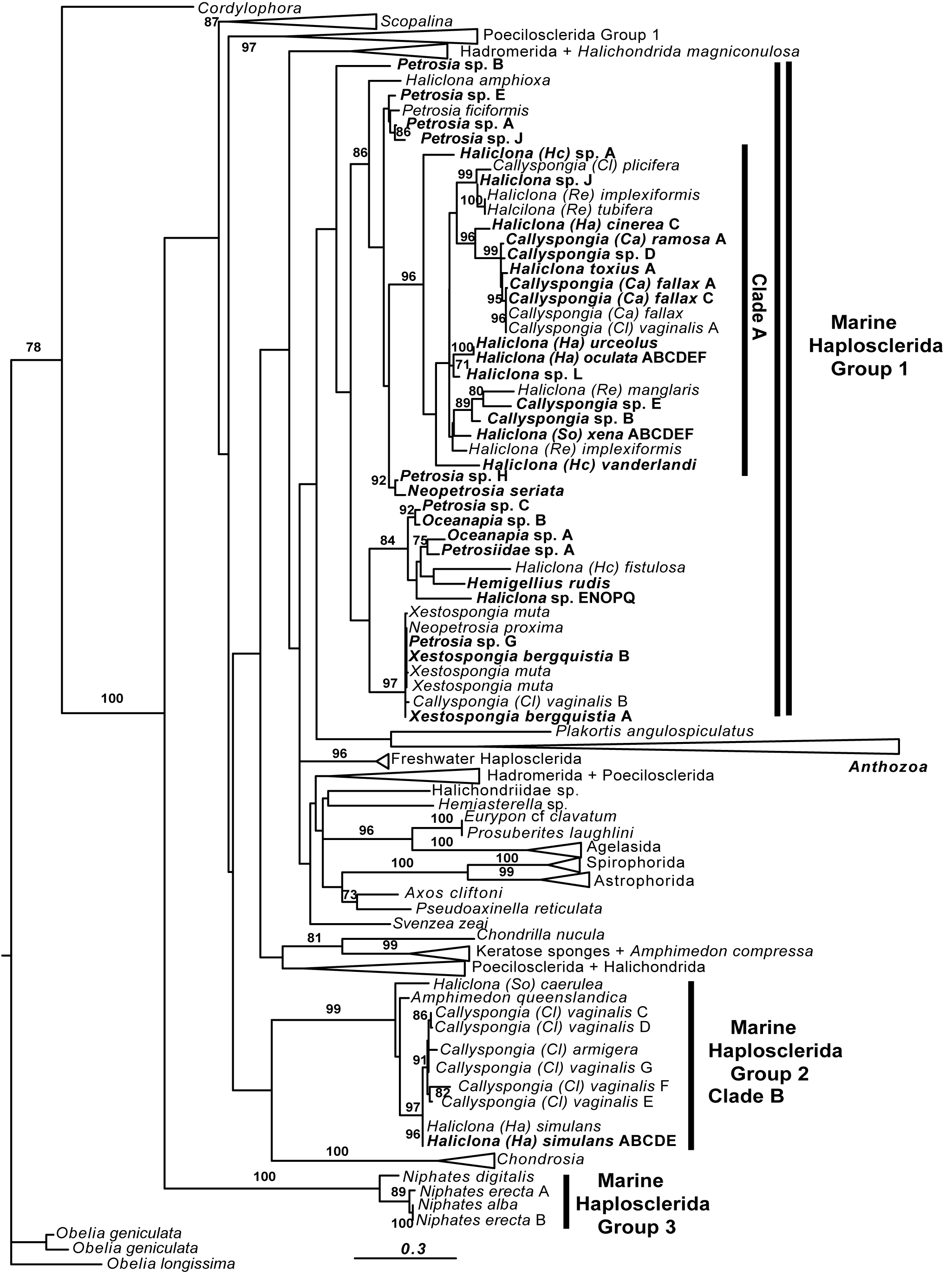

Supplement: Figure S2 — Maximum-likelihood phylogeny reconstructed from the Folmer (5′) region of the cox1 gene. The DNA substitution model parameters estimated by RAxML were; f(A) 0.26, f(C) 0.15, f(G) 0.22, f(T) 0.37; R(AC) 1.37, R(AG) 4.1, R(AT) 1.43, R(CG) 1.18, R(CT) 6.87, R(GT) 1.0; alpha 0.71; pinvar 0.41. Sequences produced during this study are in bold. Sampling locations for each taxon are given in Table S1. Other sequences were downloaded from Genbank. Numbers on the branches represent bootstrap proportions. (TIF) [file pone.0024344.s002.tif]

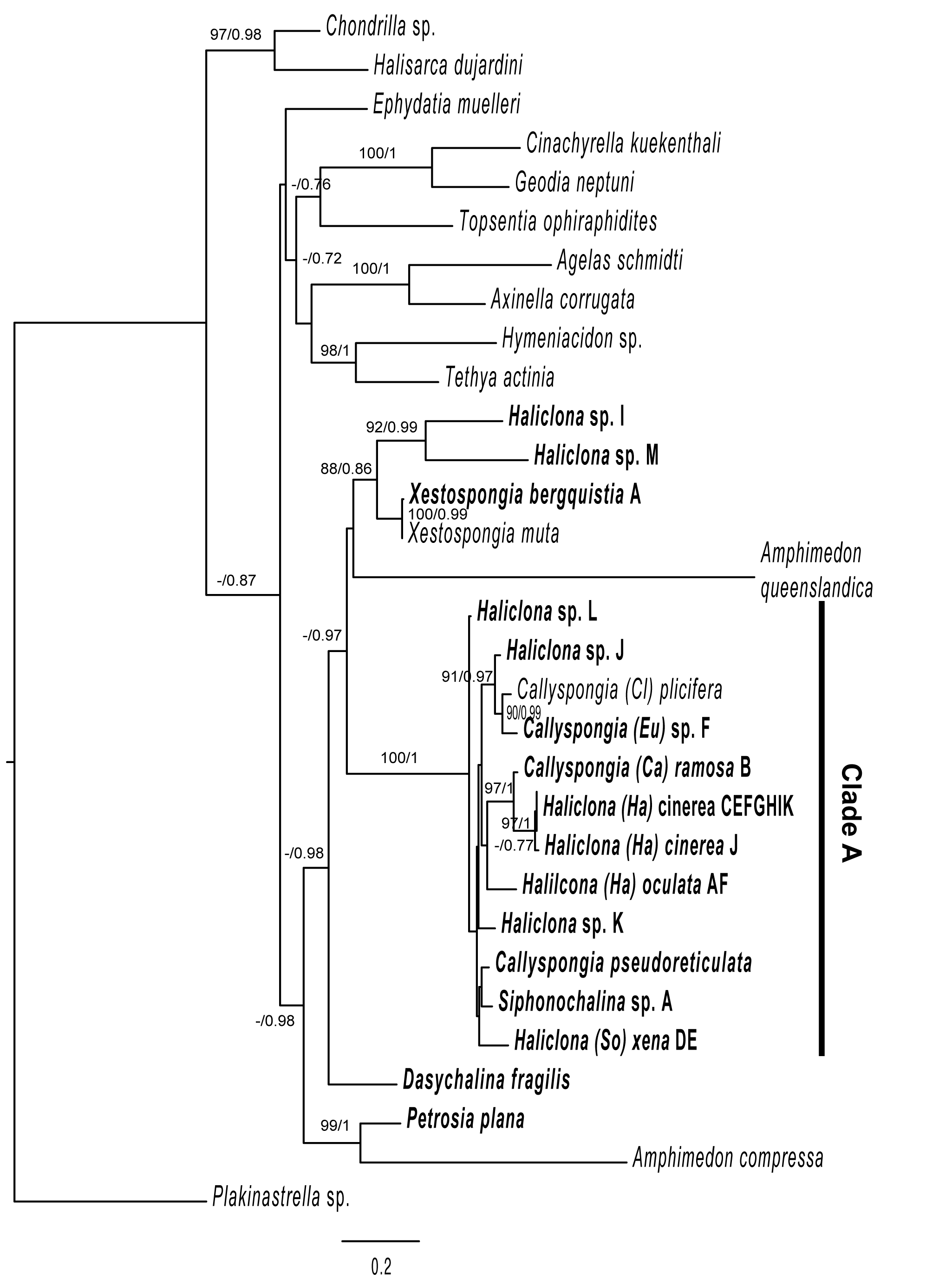

Supplement: Figure S3 — Maximum-likelihood phylogeny reconstructed from the nad1 gene. The DNA substitution model parameters by RAxML were; f(A) 0.3, f(C) 0.1, f(G) 0.21, f(T) 0.39; R(AC) 4.18, R(AG) 6.72, R(AT) 1.0, R(CG) 4.18, R(CT) 11.9, R(GT) 1.0; alpha 0.26; pinvar 0.08. Sequences produced during this study are in bold. Sampling locations for each taxon are given in Table S1. Other sequences were downloaded from Genbank (P. cf. ankodes, EU237487, X. muta, EU237490, A. queenslandica, DQ915601, C. plicifera, EU237477, A. compressa, EU237474, C. kuekenthali, EU237479, G. neptuni, AY320032, E. muelleri, EU237481, T. actinia, AY320033, A. schmidti, EU237475, A. corrugata, AY791693, H. dujardini, EU237483, Chondrilla sp., EU237478, T. ophiraphidites, EU237482). Numbers on the branches represent bootstrap proportions/posterior probabilities. (TIF) [file pone.0024344.s003.tif]
