# Supplementary material for: Phylogenetic Relationships of the Marine Haplosclerida (Phylum Porifera) Employing Ribosomal (28S rRNA) and Mitochondrial (cox1, nad1) Gene Sequence Data
Source: PLoS One. 2011 Sep 13;6(9):e24344. doi: 10.1371/journal.pone.0024344 (PMC3172223; doi:10.1371/journal.pone.0024344)
Supplement: Table S1 — List of all the marine haplosclerid specimens sequenced in this study. Sampling location, voucher number where available, and what gene regions were sequenced for each is included. (DOC) [file pone.0024344.s004.doc]

**Table S1: List of all the marine haplosclerid specimens sequenced in this study; sampling location, voucher number where available, and what gene regions were sequenced for each is included.**

**28S rRNA gene** mtCOI gene

| **Species name and Classification** | **Locality** | **Voucher** | | **D1** | **D3 - D5** | **mt Folmer** | **mt**  **NAD1** |
| --- | --- | --- | --- | --- | --- | --- | --- |
| **Suborder Haplosclerina** |  |  | |  |  |  |  |
| **Family Callyspongiidae** |  |  | |  |  |  |  |
| *Callyspongia (Ca) fallax* A | Curaçao | POR14314 | | JN178983 | JN178976 | JN242192 | **-** |
| *Callyspongia (Ca) fallax* B | Curaçao | POR14341 | | JN178984 | - | - | - |
| *Callyspongia (Ca) fallax* C | Florida | MKB219 | | - | - | JN242193 | - |
| *Callyspongia latituba* | New Zealand | MKB3144 | | JN178985 | - | - | - |
| *Callyspongia (To) multiformis* | Caribbean | POR14294 | | JN178986 | - | - | - |
| *Callyspongia (Cl) plicifera* | Curaçao | POR14276 | | JN178987 | - | - | - |
| *Callyspongia pseudoreticulata* | Indonesia | POR14552 | | JN178988 | JN178978 | - | JN242231 |
| *Callyspongia (Ca) ramosa* A | NZ | MKB3142 | | JN178989 | JN178968 | JN242194 | - |
| *Callyspongia (Ca) ramosa* B | NZ | MKB3143 | | - | - | - | JN242235 |
| *Callyspongia rosa* | PNG | MKB1031 | | JN178990 | JN178969 | - | - |
| *Callyspongia (Ca) siphonella* | Egypt | POR16627 | | JN178991 | - | - | - |
| *Callyspongia* sp. A | Oman | POR14597 | | JN178992 | JN178979 | - | - |
| *Callyspongia* sp. B | Oman | POR14635 | | JN178993 | JN178974 | JN242195 | - |
| *Callyspongia* sp. C | Oman | POR14599 | | JN178994 | JN178981 | - | - |
| *Callyspongia* sp. D | Oman | NA | | - | - | JN242196 | **-** |
| *Callyspongia* sp. E | PNG | MKB1618 | | JN178995 | - | JN242197 | **-** |
| *Callyspongia (Eu)* sp. F | PNG | MKB1668 | | JN178996 | JN178970 | - | JN242233 |
| *Siphonochalina* sp. A | Oman | POR14630 | | JN178997 | JN178980 | - | JN242232 |
| *Siphonochalina* sp. B | PNG | NA | | JN178998 | - | - | - |
|  |  |  | |  |  |  |  |
| **Family Chalinidae** |  |  | |  |  |  |  |
| *Chalinula hooperi* | Indonesia | POR17651 | | JN178999 | JN178956 | - | - |
| *Chalinula limbata* | Ireland | MIIG0252 | | JN179000 | JN178951 | - | - |
| *Cladocroce* sp. | Indonesia | POR14529 | | JN179001 | - | - | - |
| *Haliclona (Ha) cinerea* A | France | POR14139 | | JN179002 | JN178966 | - | - |
| *Haliclona (Ha) cinerea* B | France | POR14110 | | JN179003 | JN178967 | - | - |
| *Haliclona (Ha) cinerea* C | France | POR14138 | | B | B | JN242198 | JN242236 |
| *Haliclona (Ha) cinerea* D | Ireland | Mc3344 | | B | - | B | - |
| *Haliclona (Ha) cinerea* E | Ireland | MIIG0107 | | - | - | - | B |
| *Haliclona (Ha) cinerea* F | Ireland | MIIG0154 | | - | - | - | B |
| *Haliclona (Ha) cinerea* G | Ireland | MIIG0060 | | - | - | - | B |
| *Haliclona (Ha) cinerea* H | Ireland | MIIG0207 | | - | - | - | B |
| *Haliclona (Ha) cinerea* I | Ireland | MIIG0232 | | - | - | - | B |
| *Haliclona (Ha) cinerea* K | Ireland | MIIG0166 | | - | - | - | B |
| *Haliclona (Ha) cinerea* J | Ireland | MIIG0233 | | - | - | - | JN242237 |
| *Haliclona (Re) fascigera* | Micronesia | MKB150 | | JN179004 | JN178973 | - | - |
| *Haliclona (Ge) fibulata* | Ireland | MIIG00256 | | JN179005 | JN178950 | - | - |
| *Haliclona koremella* | PNG | MKB1097 | | JN179006 | JN178971 | - | - |
| *Haliclona (Re) manglaris* | BDT | SI06_225 | | - | JN178948 | **-** | **-** |
| *Haliclona (Ha) oculata* A | France | POR14116 | | JN179007 | JN178975 | JN242199 | JN242230 |
| *Haliclona (Ha) oculata* B | Holland | HAP221 | | B | - | B | - |
| *Haliclona (Ha) oculata* C | Netherlands | HAP239 | | B | - | B | - |
| *Haliclona (Ha) oculata* D | Netherlands | HAP240 | | B | - | B | - |
| *Haliclona (Ha) oculata* E | Germany | HAP260 | | B | - | B | - |
| *Haliclona (Ha) oculata* F | Germany | HAP263 | | B | - | B | B |
| *Haliclona (Ha) simulans* A | Ireland | Mc3342 | | JN179008 | - | JN242200 | - |
| *Haliclona (Ha) simulans* B | Ireland | MIIG0065 | | B | - | B | - |
| *Haliclona (Ha) simulans* C | Ireland | MIIG0066 | | B | - | B | - |
| *Haliclona (Ha) simulans* D | Ireland | MIIG0067 | | B | - | B | - |
| *Haliclona (Ha) simulans* E | Ireland | MIIG0002 | | - | - | JN242201 | **-** |
| *Haliclona (Hc)* sp. A | Caribbean | MKB550 | | JN179009 | JN178965 | JN242202 | **-** |
| *Haliclona* sp. B | Oman | POR14615 | | JN179010 | - | - | - |
| *Haliclona* sp. C | Ireland | Mc3335 | | JN179011 | - | - | - |
| *Haliclona* sp. E | Ireland | Mc3343 | | JN179012 | - | JN242210 | - |
| *Haliclona* sp. N | Ireland | Mc3333 | | - | - | B | - |
| *Haliclona* sp. O | Ireland | Mc3338 | | - | - | B | - |
| *Haliclona* sp. P | Ireland | Mc3345 | | - | - | B | - |
| *Haliclona* sp. Q | Ireland | MIIG235 | | - | - | B | - |
| *Haliclona* sp. F | NA | ORK 3032 | | JN179013 | - | - | - |
| *Haliclona* sp. G | NA | N423 | | - | - | Y | **-** |
| *Haliclona* sp. H | Ireland | MIIG0258 | | JN179014 | - | - | - |
| *Haliclona* sp. I | Ireland | MIIG0193 | | - | - | - | JN242225 |
| *Haliclona* sp. J | Ireland | MIIG0113 | | - | - | JN242203 | JN242234 |
| *Haliclona* sp. K | Ireland | MIIG0212 | | - | - | - | JN242239 |
| *Haliclona* sp. L | Ireland | MIIG0210 | | - | - | JN242204 | JN242238 |
| *Haliclona* sp. M | Ireland | MIIG0194 | | - | - | JN242205 | JN242240 |
| *Haliclona (Ge) toxius* | Oman | POR14642 | | JN179015 | - | JN242206 | - |
| *Haliclona (Ha) urceolus* | Ireland | MIIG0223 | | - | - | JN242207 | **-** |
| *Haliclona (Hc) vanderlandi* | Indonesia | POR14497 | | JN179016 | - | JN242208 | **-** |
| *Haliclona (Hc) vansoesti* A | Curaçao | POR14240 | | JN179017 | - | - | - |
| *Haliclona (Hc) vansoesti* B | France | POR14229 | | B | - | - | - |
| *Haliclona (So) vermeuleni* | BDT | SI06_269 | | - | JN178947 | - | - |
| *Haliclona (So) walentinae* | BDT | SI06_216 | | - | JN178946 | - | - |
| *Haliclona (So) xena* A | Netherlands | HAP244 | | JN179018 | - | JN242209 | - |
| *Haliclona (So) xena* B | Holland | HAP222 | | B | - | B | - |
| *Haliclona (So) xena* C | Holland | HAP223 | | B | - | B | - |
| *Haliclona (So) xena* D | Netherlands | HAP241 | | B | - | B | B |
| *Haliclona (So) xena* E | Netherlands | HAP242 | | B | - | B | JN242229 |
| *Haliclona (So) xena* F | Netherlands | HAP243 | | B | - | B | - |
|  |  |  | |  |  |  |  |
| **Family Niphatidae** |  |  | |  |  |  |  |
| *Amphimedon compressa* | BDT | SI06_25 | | - | JN178945 | - | - |
| *Amphimedon paraviridis* | Indonesia | POR17685 | | JN179019 | JN178952 | - | - |
| *Amphimedon* sp. | Indonesia | POR14524 | | JN179020 | - | - | - |
| *Amphimedon viridis* | Indonesia | POR14528 | | JN179021 | JN178954 | - | - |
| *Cribrochalina* sp. | PNG | MKB1023 | | - | JN178957 | - | - |
| *Cribrochalina vasculum* | Caribbean | MKB538 | | JN179022 | JN178972 | - | - |
| *Dasychalina fragilis* | Indonesia | POR14455 | | JN179023 | - | - | JN242226 |
| *Gelliodes fibulata* | PNG | MKB1026 | | - | - | - | - |
| *Hemigellius rudis* | Antarctica | POR13247 | | - | - | JN242211 | **-** |
| *Niphates elegans* | PNG | MKB1034 | | JN179024 | - | - | - |
| *Niphates erecta* | BDT | SI06_279 | | - | JN178944 | - | - |
| *Niphates olemda* | Indonesia | POR14449 | | JN179025 |  | - | - |
| *Niphates* sp. A | Indonesia | POR14462 | | JN179026 | JN178958 | - | - |
| *Niphates* sp. B | Micronesia | MKB148 | | JN179027 | JN178959 | - | - |
| *Pachychalina* sp. | Indonesia | POR14493 | | JN179028 | - | - | - |
|  |  |  | |  |  |  |  |
| **Suborder Petrosina** |  |  | |  |  |  |  |
| **Family Petrosiidae** |  |  | |  |  |  |  |
| *Petrosiidae* sp. A | New Zealand | KAH9907/48 | | - | - | JN242212 | **-** |
| *Petrosiidae* sp. B | Norfolk Rise | MKB1785 | | JN179029 | - | - | - |
| *Acanthostrongylophora ingens* | Indonesia | POR17500 | | JN179030 | JN178955 | - | - |
| *Neopetrosia seriata* | PNG | MKB1048 | | JN179031 | - | JN242213 | **-** |
| *Neopetrosia tuberosa* | Kenya | POR17660 | | JN179032 | - | - | - |
| *Petrosia (Petrosia) hoeksemai* A | Indonesia | POR14474 | | JN179033 | JN178961 | - | - |
| *Petrosia (Petrosia) hoeksemai* B | Indonesia | POR14517 | | B | B | - | - |
| *Petrosia (Petrosia) plana* | Indonesia | POR14516 | | JN179034 | - | - | JN242227 |
| *Petrosia* sp. A | Caribbean | MKB557 | | - | - | JN242214 | **-** |
| *Petrosia* sp. B | Caribbean | MKB560 | | JN179035 | - | JN242215 | **-** |
| *Petrosia* sp. C | Fiji | MKB983 | | - | - | JN242216 | **-** |
| *Petrosia* sp. D | PNG | MKB1020 | | JN179036 | JN178962 | - | - |
| *Petrosia* sp. E | PNG | MKB1028 | | JN179037 | - | JN242217 | - |
| *Petrosia* sp. F | PNG | MKB1068 | | JN179038 | JN178960 | - | - |
| *Petrosia* sp. G | PNG | MKB1634 | | - | - | JN242218 | **-** |
| *Petrosia (Strongylophora)* sp. H | Caribbean | MKB587 | | JN179039 | - | JN242219 | - |
| *Petrosia* sp. J | New Caledonia | | NA | - | - | JN242220 | - |
| *Petrosia strongylata* | Indonesia | POR16747 | | JN179040 | - | - | - |
| *Xestospongia bergquistia* A | Indonesia | POR14457 | | - | - | JN242221 | JN242228 |
| *Xestospongia bergquistia* B | PNG | MKB1663 | | - | - | JN242222 | **-** |
|  |  |  | |  |  |  |  |
| **Family Phloeodictyidae** |  |  | |  |  |  |  |
| *Aka mucosa* | Caribbean | MKB535 | | JN179041 | JN178963 | - | - |
| *Aka* sp. | NA | SI06_219 | | - | JN178949 | - | - |
| *Calyx* sp. | PNG | MKB1132 | | JN179042 | JN178977 | - | - |
| *Oceanapia* sp. A | Indonesia | POR14507 | | - | - | JN242223 | **-** |
| *Oceanapia* sp. B | Caribbean | MKB586 | | JN179043 | JN178964 | JN242224 | **-** |
| *Tabulocalyx* sp. | NA | POR17655 | | JN179044 | JN178953 | - | - |
|  |  |  | |  |  |  |  |
| **Suborder Spongillina** |  |  | |  |  |  |  |
| *Nudospongilla* sp. |  |  | | JN179045 | JN178982 |  |  |
| *Spongillina* sp. |  |  | | JN179046 |  |  |  |
|  |  |  | |  |  |  |  |

Species are organised according to the morphological classification [2]. Genbank accession numbers are listed for all sequences generated in this study and included in the analyses. B indicates identical sequences. Vouchers begining with POR refer to those from the University of Amsterdam, those with MKB, NIWAKD and KAH are from MK’s personal collection, those with MIIG and HAP are held at the Molecular Evolution & Systematics Laboratory at National University of Ireland Galway, those with Mc are held at the Museum of Northern Ireland, those with SI06 are at the NMNH, Smithsonian Institution. Subgenera of *Callyspongia* and *Haliclona* are included in brackets: *Ca = Callyspongia, Cl = Cladochalina, Eu = Euplacella, To = Toxochalina, Ge = Gellius, Ha = Haliclona, Hc = Halichoclona, Re = Reniera, So = Soestella*. For locality PNG = Papua New Guinea, BDT = Bocas del Toro, Panama, NA = not available.
