# Supplementary material for: Phylogenetic Relationships of the Marine Haplosclerida (Phylum Porifera) Employing Ribosomal (28S rRNA) and Mitochondrial (cox1, nad1) Gene Sequence Data
Source: PLoS One. 2011 Sep 13;6(9):e24344. doi: 10.1371/journal.pone.0024344 (PMC3172223; doi:10.1371/journal.pone.0024344)
Supplement: Table S2 — Primer sequence information for each primer used in amplifying each gene region. (DOC) [file pone.0024344.s005.doc]

**Table S2: Primer sequence information for each primer used in amplifying each gene region.**

|  | **5’ → 3’ sequence** | **Reference** |
| --- | --- | --- |
| **28S rRNA** |  |  |
| **D1 region** |  |  |
| LSU5F | TAGGTCGACCCGCTGCCYTTAAGC | Olsen (NHM London,Pers. Comm) |
| LSU300R | CAACTTTCCCTCACGGTACTT | “ |
|  |  |  |
| **D2 region** |  |  |
| LSU300F | CAAGTACCGTGAGGGAAAGTT | Olsen (NHM London,Pers. Comm) |
| LSU1200R | GCATAGTTCACCATCTTTCGG | “ |
|  |  |  |
| **D3-D5 region** |  |  |
| LSU900F | CCGTCTTGAAACACGGACCAA | Olsen (NHM London,Pers. Comm) |
| LSU1642R | CCAGCGCCATCCATTTTC | “ |
|  |  |  |
| ***cox1*** |  |  |
| coxF3 | GGTCAACAAATCATAAAGATATTGG | Folmer et al. 1994 |
| coxR3 | TAAACTTCAGGGTGACCAAAAAATCA | “ |
|  |  |  |
| ***nad1*** |  |  |
| nad1SF2 | GCYGATGGATTAAAATTATTYACTAAAG | This study |
| nad1SR1 | CCTTTCGTTCYGCYAAWGTTAAATA | “ |
